# Fovea-threatening and fovea-involving peripheral Coats disease: effects of posture and intervention

**DOI:** 10.1186/s40942-022-00382-4

**Published:** 2022-06-17

**Authors:** Eduardo Cunha de Souza, Evandro Rosa, João Rafael de Oliveira Dias, Fernando Korn Malerbi, Bruno Campelo Leal, Helio Paulo Primiano Junior

**Affiliations:** 1 Faculdade de Medicina da Universidade de São Paulo, Av. Dr. Arnaldo, 455, São Paulo, 01246-903 Brazil; 2 Hospital de Olhos Sadalla Amin Ghanem, Joinville, Brazil; 3 Universidade Federal de São Paulo, São Paulo, Brazil; 4 Faculdade de Medicina da Unit de Sergipe, Aracaju, Brazil

## Abstract

**Background:**

We believe that our experience with patients presenting with Coats disease and macular sparing should be shared with our colleagues. We would like to show the effect of posture and prompt intervention in cases with fovea-threatening and/or fovea-involving peripheral Coats disease (FTPCD). This association has been poorly debated in our specialty and literature. We call the attention for the unexpexted scenario of observing the lost of the fovea during some types of traditional and prompt interventional treatments of these cases with previous 20/20 vision (something that we have been studying and observing for many years). In order to publish our best representative cases, we have chosen 8 Brazilian patients (age range, 7–62 years; 5 male) with FTPCD. All patients underwent multimodal imaging and different treatments (observation, sleep-posture repositioning, laser, intraocular steroids, and/or anti-vascular endothelial growth factor therapy). All patients, initially, informed to adopt a sleeping lateral-down position, favoring exudation shifting to the fovea pre-treatment. Most promptly-treated patients in this way (n = 4), developed subretinal fluid and exudates in the macula and some had irreversible central visual loss (n = 3). Patients with recent fovea-involving exudation who changed postural sleep position (to protect the foveal area) before and during treatment fared better, with some preserved central vision and an intact fovea (n = 5). The fundus status was correlated with the gravitational effects of posture before and after treatment. Despite prepared as an observational/interventional study, with a small number of cases, the most difficult part is documenting the sleep position of these patients and its influence in the outcomes as there is not good way to prove how well or poorly the positioning occurred in our cases. Finally, we also intended to call the attention to the fact that Coats disease must be studied in all its clinical stage variants and not only seen as a potential blinding and incurable ocular disease.

**Case presentation:**

This study is a retrospective and/or interventional analysis of eight cases with a less severe clinical variant of classic Coats disease that we refer to as fovea-threatening and fovea-involving peripheral Coats disease (FTPCD).

All cases were unilateral with no systemic disease or family history of Coats disease. The bilateral anterior segment and intraocular pressure were normal in all patients. The characteristics of all patients are shown in the Table.

**Conclusion:**

The funduscopic features of FTPCD are fundamental to disease understanding and optimal management. Habitual posturing may affect the fundus morphologic features of retinal exudation as observed in all current patients with exudative peripheral Coats disease. When sleep habitual posture is not observed in patients with FTPCD, the effects of prompt invasive treatments can cause rapid visual loss because of foveal subretinal pooling of exudates post-treatment. Initial vigilant adjusting of the habitual sleep posture for several patients with FTPCD, before the indication of traditional invasive treatments (laser and/or pharmacologic medications) can result in improved vision and fundoscopic morphologic features.

**Supplementary Information:**

The online version contains supplementary material available at 10.1186/s40942-022-00382-4.

## Introduction

Most patients with classic Coats disease, predominantly young men, present with extensive exudation and a total retinal detachment. The resulting poor visual acuity (VA), ocular pain, glaucoma, leukocoria, and squinting are definitive findings that occur within 10 years after birth in 60% to 70% of cases [1, 2]. The clinical disease stages can be classified according to Shields’s criteria [3], with stage 1 involving only retinal telangiectasia and stage 2 telangiectasia and exudation, with 2A representing extrafoveal exudation and stage 2B exudation involving the fovea. Daruich et al. [4] suggested an updated classification introducing two subcategories within stage 2B, i.e., stage 2B1 without subfoveal nodules and stage 2B2 with subfoveal nodules; stage 3 exudative retinal detachment, in which stage 3A1 does not involve the fovea, stage 3A2 involves the fovea, and stage 3B involves a total retinal detachment; stage 4 total retinal detachment with glaucoma; and stage 5 severe end-stage disease. The specific cause of Coats disease is unknown; however, one theory is that a somatic mutation of the *Norrie disease protein* gene leads to Coats disease. This gene plays a vital role in retinal blood vessel development [5]. Nevertheless, it is unclear which factors determine the extent of the retinal involvement, progression, and quality of exudation in cases first diagnosed with the disease [1–3]. To better understand the results of the characteristic fundus response in these cases, in 1886, Starling [6] hypothesized about capillary interstitial fluid transfer in humans, in which the capillaries were considered impermeable to plasma proteins. He postulated that the flow of fluid across the capillary wall depended on a balance between the hydrostatic pressure and osmotic pressure of plasma proteins within its lumen [6]. Thus, at the retinal level, because the arterial end of the capillary lumen pressure is greater than the oncotic pressure, fluid is pushed out, and at the venous end, due to higher osmotic pressure, fluid is withdrawn into the capillary lumen. All current patients presented with extensive exudation threatening or recently involving the foveal area (VA range, 20/20−20/200). According to the Starling principle, the peripheral Coats abnormal vessels were the source of leaking serum and plasma proteins in these patients due to inner retinal barrier damage. The current study shows that an interaction between gravitational positioning and physiologic forces (normal retinal capillaries and retinal pigment epithelium/choriocapillaries complex) may explain the funduscopic features of these patients before and after treatment [7–9].

### Case 1

**Fig. 1:**
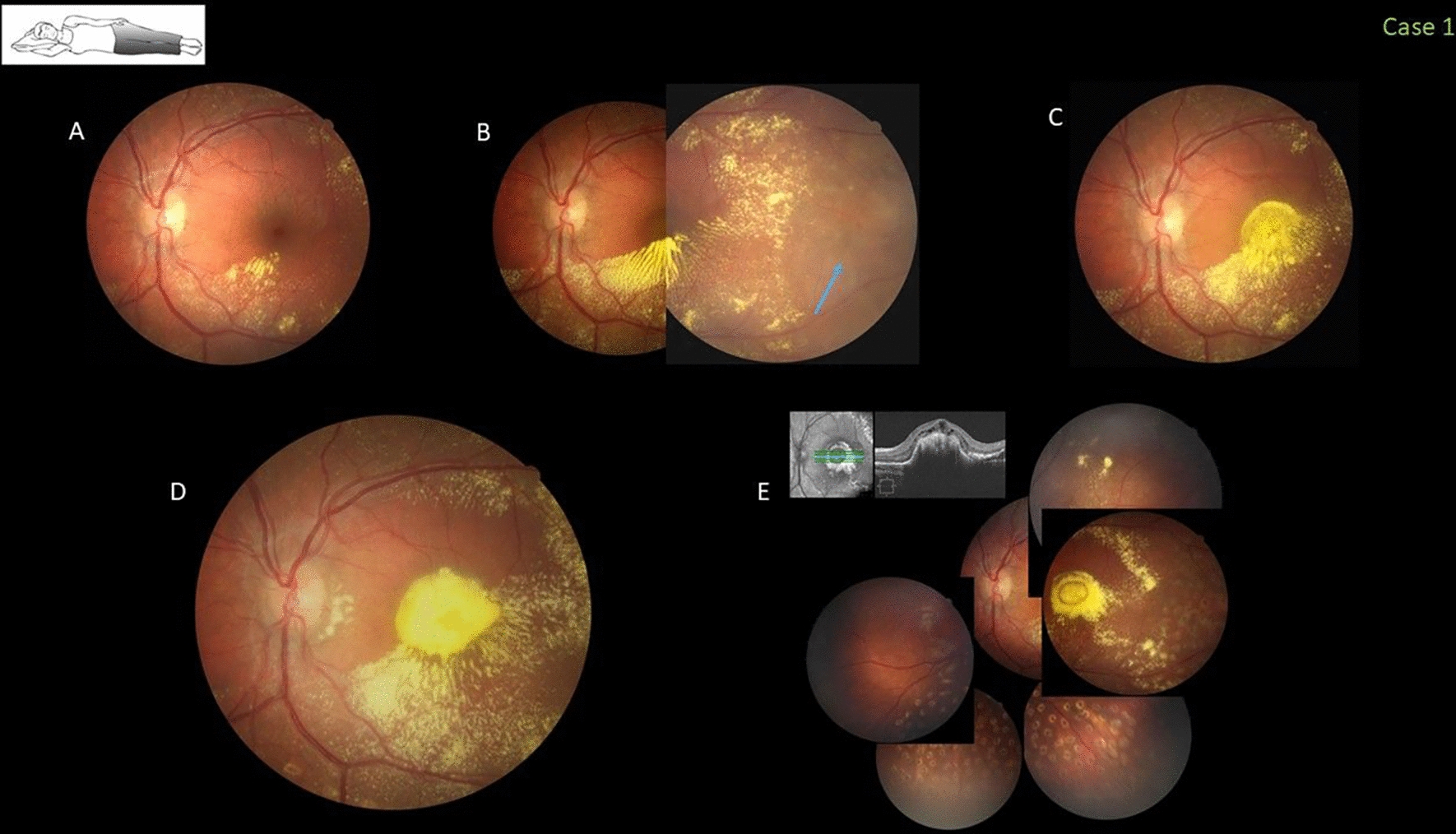
Case 1. Fovea-threatening peripheral Coats disease in a 7-year-old boy who slept in the nasal-down position (inset). **A** Four weeks after the first indirect argon green laser treatment, the visual acuity (VA) was 20/20. **B** Four weeks after complementary slit-lamp argon green laser plus a 1.25-mg intravitreal bevacizumab injection, reactive peripheral subretinal fluid is seen (arrow). **C** The VA decreased to 20/200 with pooling of exudates in the fovea that advanced centripetally with subretinal fluid after a second bevacizumab injection 4 weeks later. **D, E** The extended follow-up period confirmed sequentially the macular area with progressive chronic pooling of subretinal fluid and exudates with a fibrotic subretinal nodule (stage 2B2) and irreversible central visual loss to counting fingers

This healthy 7-year-old boy with 20/20 bilateral vision was referred for evaluation of leukocoria in his left eye. Fundus examination showed abnormal telangiectatic and lightbulb vessels along the temporal and inferior periphery associated with extensive lipid exudation threatening the foveal area. A characteristic gravitational line of lipid precipitation was observed inferiorly. Stage 2A Coats disease was diagnosed and prompt indirect laser treatment (Nd-YAG 532) was applied as the only initial procedure. After 4 weeks of laser treatment, the VA decreased to 20/200 because of pooling of concentrated exudates that migrated into the fovea (stage 2B1). The patient then was treated with complementary slit-lamp argon green laser plus a 1.25-mg injection of intravitreal bevacizumab (Avastin, Genentech Inc., South San Francisco, CA). Four weeks later, the foveal exudation and subretinal fluid expanded further with formation of a fibrotic nodular reaction after a second injection of bevacizumab (Fig. 1). The family later confirmed that the patient used to sleep in the nasal-down position even during all treatments, which supported the pooling of exudates in the foveal area.

### Case 2

**Fig. 2:**
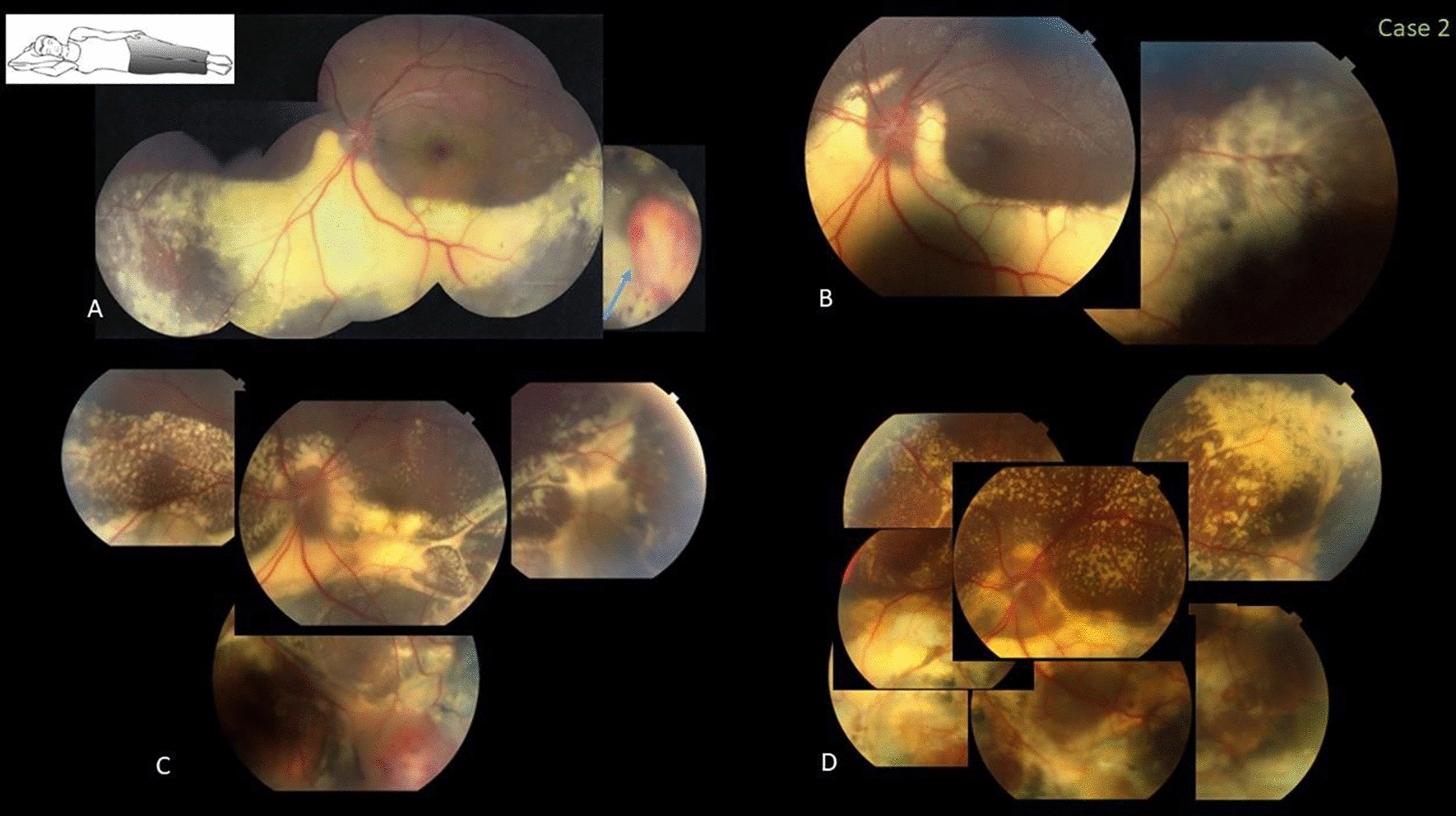
Case 2. Fovea-threatening peripheral Coats disease in an 11-year-old girl who slept in the nasal-down position (inset). **A** A color fundus image when the visual acuity (VA) was 20/20 obtained 4 years previously (2010) already showed extensive inferior gravitated exudation and an apparent active vasoproliferative-like lesion (arrow). **B** A color fundus image obtained when the patient was first seen to evaluate leukocoria (2014); the VA was 20/20 (observation was recommended). **C, D** Color fundus images of the follow-up after prompt treatment show the macular area and surrounding retina invaded by aggressive exudation and formation of areas with subretinal fibrosis and irreversible loss of central vision to counting fingers

In 2014, this healthy 11-year-old girl with 20/20 vision bilaterally was referred for evaluation of leukocoria in the left eye. Fundus examination of that eye showed abnormal telangiectatic vessels associated with large, isolated vasoproliferative tumor-like lesions along the temporal and inferior periphery with extensive lipid exudation threatening the foveal area. A characteristic gravitational line of lipid precipitation was observed inferiorly, from the nasal to temporal midperipheral retina. Stage 2A Coats disease was diagnosed, and based on the examination and color family photographs from 2010, preserved central vision (20/20), and the chronic protected status of the foveal area, she was observed initially. However, the family consulted another institution where she was treated promptly with indirect laser (Nd-YAG 532) and subsequent 1.25-mg intravitreal injections of bevacizumab. The VA decreased progressively to counting fingers due to extensive exudative and fibrotic destruction of the foveal area (Fig. 2). The family later confirmed that the patient slept in a nasal-down position.

### Case 3

**Fig. 3:**
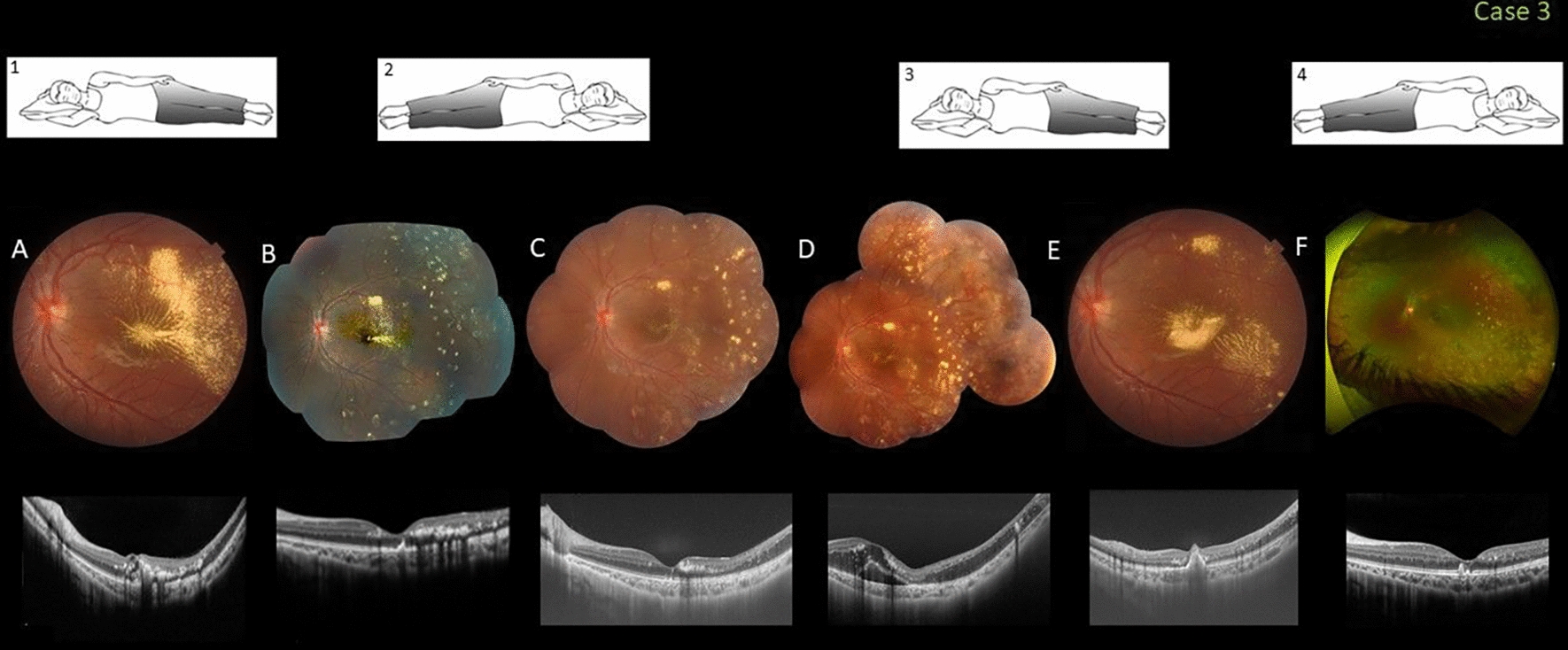
Case 3. Fovea-involving peripheral Coats disease in an 11-year-old boy who slept in the nasal-down position (inset 1). **A** A color fundus image when the visual acuity (VA) was 20/100 shows extensive temporal peripheral exudation invading the fovea. A spectral-domain optical coherence tomography (SD-OCT) image shows the intraretinal and subfoveal hyperreflectivity of the heavy exudation. **B** Eight weeks after the patient changed his sleep position (inset 2), followed by a series of laser treatments and intraocular anti-vascular endothelial growth factor (VEGF) injections, partial resolution of the exudation is confirmed by SD-OCT. The VA improved to 20/40. **C** Three months after the first presentation, the exudation is almost completely resolved. The VA was 20/25. **D** After 3 months, a new episode of foveal invasion with pooling of concentrated exudates is seen; the VA was 20/150 when the patient was not sleeping in the lateral-down position (inset 3). **E** He was lost to follow-up for 4 to 6 months and returned again with heavy foveal exudation; the VA was 20/200. **F** Six months after the sleep position was adjusted (inset 4), complementary laser, and monthly anti-VEGF injections, the exudation and vision improved to 20/30

This healthy 11-year-old boy was referred for evaluation of central visual loss in the left eye to 20/100. Fundus examination of that eye showed abnormal extensive peripheral telangiectatic and lightbulb capillaries associated with lipid exudation affecting the foveal area. He was diagnosed with stage 2B1 Coats disease. The patient slept in the nasal-down position that facilitated pooling of the exudates in the foveal area. Prompt repositioning to a temporal-down position during sleep was recommended for the first 4 weeks. After that, a series of laser treatments and intraocular anti-vascular endothelial growth factor (VEGF) injections were planned for the patient. The foveal exudation progressively improved and the VA improved to 20/25. After 3 months, pooling of concentrated exudates in the fovea recurred (stage 2B1). The family confirmed that the patient was not sleeping in a position that prevented accumulation of exudates. The patient was again advised to change his sleep position and to continue complementary laser and monthly injections of anti-VEGF therapy. He was lost to follow-up for 4 to 6 months and returned again with heavy foveal exudation; the VA was 20/200. Six months after the sleep position was adjusted (inset 4), complementary laser, and monthly anti-VEGF injections, the exudation and vision improved to 20/30 (Fig. 3).

### Case 4

**Fig. 4:**
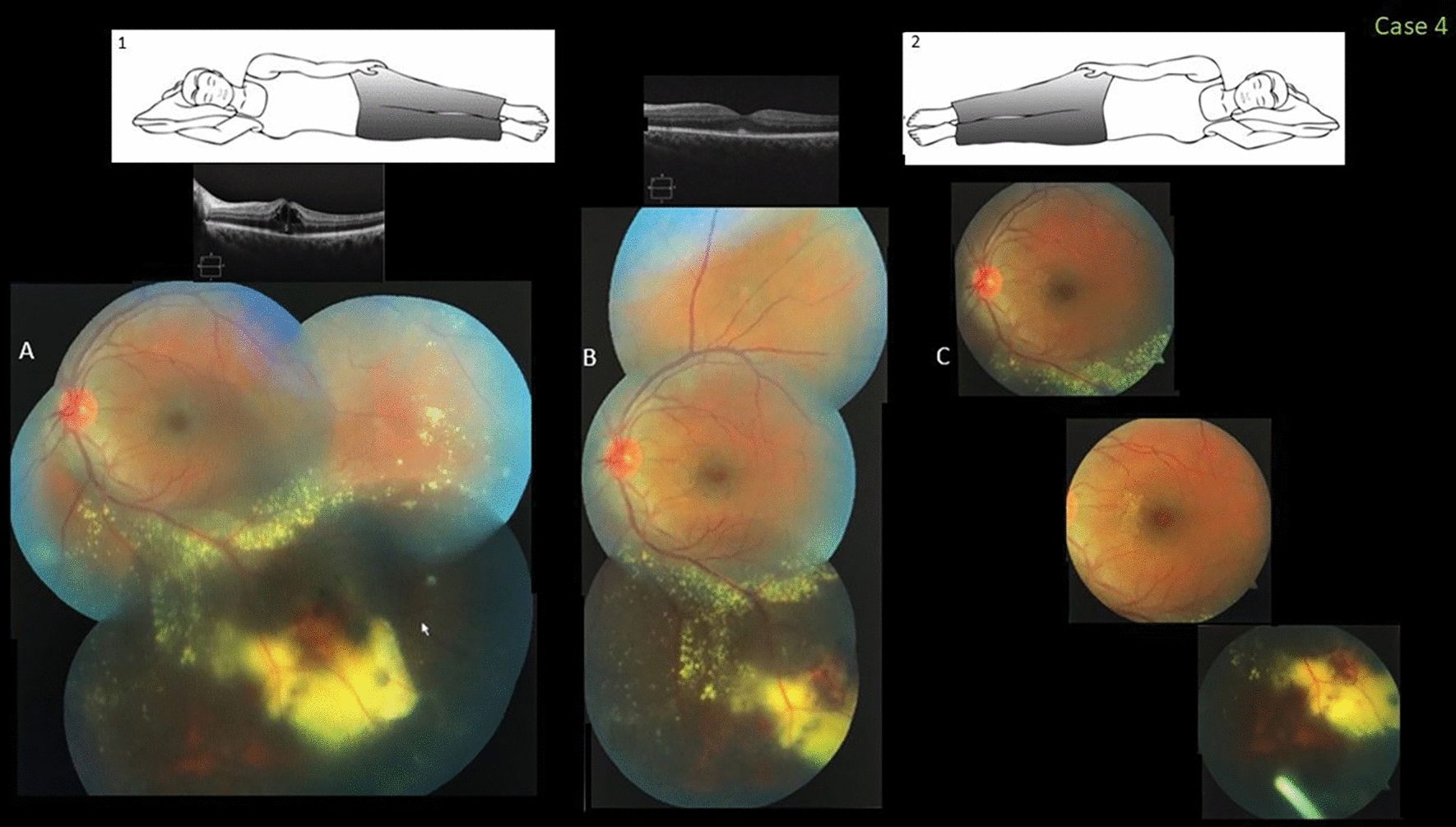
Case 4. Fovea-involving peripheral Coats disease in a 34-year-old man who slept in the nasal-down position with a flat pillow (inset 1). **A** A color fundus image obtained when the visual acuity (VA) was 20/100 shows inferotemporal peripheral exudative retinal vascular abnormalities and an exudative retinal detachment recently affecting the fovea. A spectral-domain optical coherence tomography (SD-OCT) image shows a foveal serous detachment. **B** Four weeks after the initial observation, changing the sleep position to a lateral-down high position (inset 2) and raising the head of the bed resulted in resolution of the foveal detachment. The VA returned to 20/30. **C,** Four weeks after the first intravitreal injection of Ozurdex, the foveal exudation is coming under control

A healthy 34-year-old man was referred for a second opinion and management after presenting with central visual blurring in the left eye. The VA was 20/100 and fundus examination showed abnormal exudative telangiectatic vessels along the temporal and inferior periphery associated with an exudative retinal detachment that threatened the foveal area. Stage 2B Coats disease was confirmed. Because the patient slept on a flat pillow in a nasal-down position that facilitated exudative accumulation, a prompt recommendation was made to raise the head of the bed and for the patient to sleep in a lateral-down sleep position. Four weeks later, the foveal detachment surprisingly resolved. The VA returned to 20/30 and the patient was treated with a first intravitreal injection of the Ozurdex implant (dexamethasone intravitreal implant, Allergan, Irvine, CA) when he showed progressive control of the foveal exudation (Fig. 4). He was ultimately lost to follow-up.

### Case 5

**Fig. 5:**
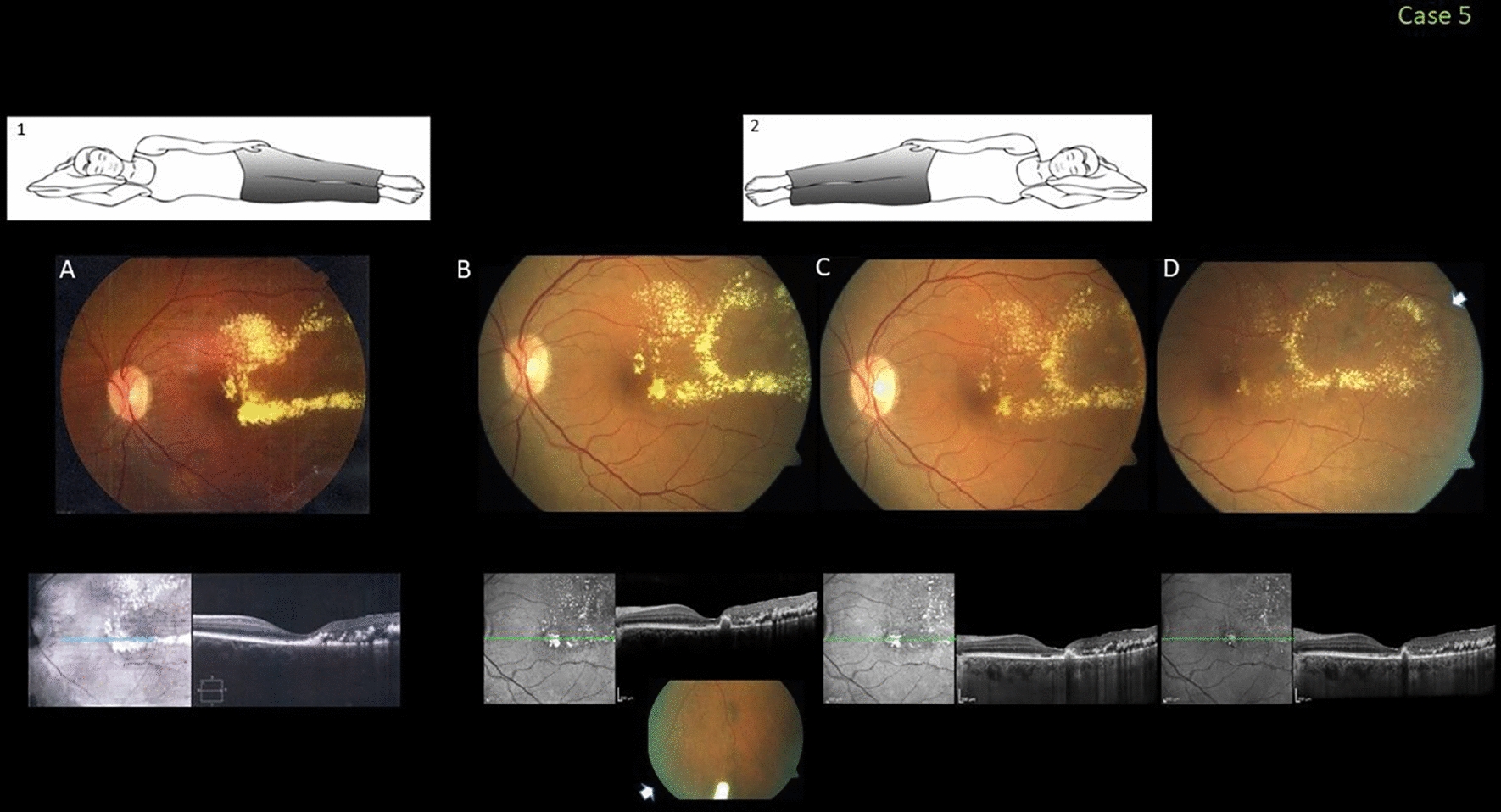
Case 5. Fovea-involving peripheral Coats-like disease in a 62-year-old woman who slept in the lateral-nasal position (inset 1). **A** A color fundus image shows the chronic temporal peripheral exudation recently affecting the fovea; the visual acuity (VA) was 20/150. A spectral-domain optical coherence tomography (SD-OCT) image shows the foveal and temporal intraretinal hyperreflective exudates. **B–D** Images obtained at 8, 16, and 24 weeks, respectively, after the first change in the patient’s sleep position to the lateral-down high position (inset 2), followed by one intravitreal injection of Ozurdex (arrowhead) and laser treatment. The VA improved to 20/40. SD-OCT shows progressing partial resolution of the exudation

This healthy 62-year-old woman with no systemic disease was referred for evaluation of chronic central visual loss in the left eye (VA, 20/100). Fundus examination of that eye showed abnormal telangiectatic and lightbulb capillaries confined to the equatorial area associated with extensive lipid exudation affecting the foveal area. Coats-like Leber’s miliary aneurysms were diagnosed. Considering the chronicity and risk of prompt treatment-related foveal deterioration in this case, because the patient slept in a nasal-down position that facilitated exudation, repositioning during sleeping to the left temporal side was recommended. Four weeks after, the foveal exudation had resolved partially and the VA improved to 20/50. Further involution of the exudation along with improved vision to 20/40 occurred after injection of the Ozurdex implant and one application of argon laser treatment (Fig. 5).

### Case 6

**Fig. 6:**
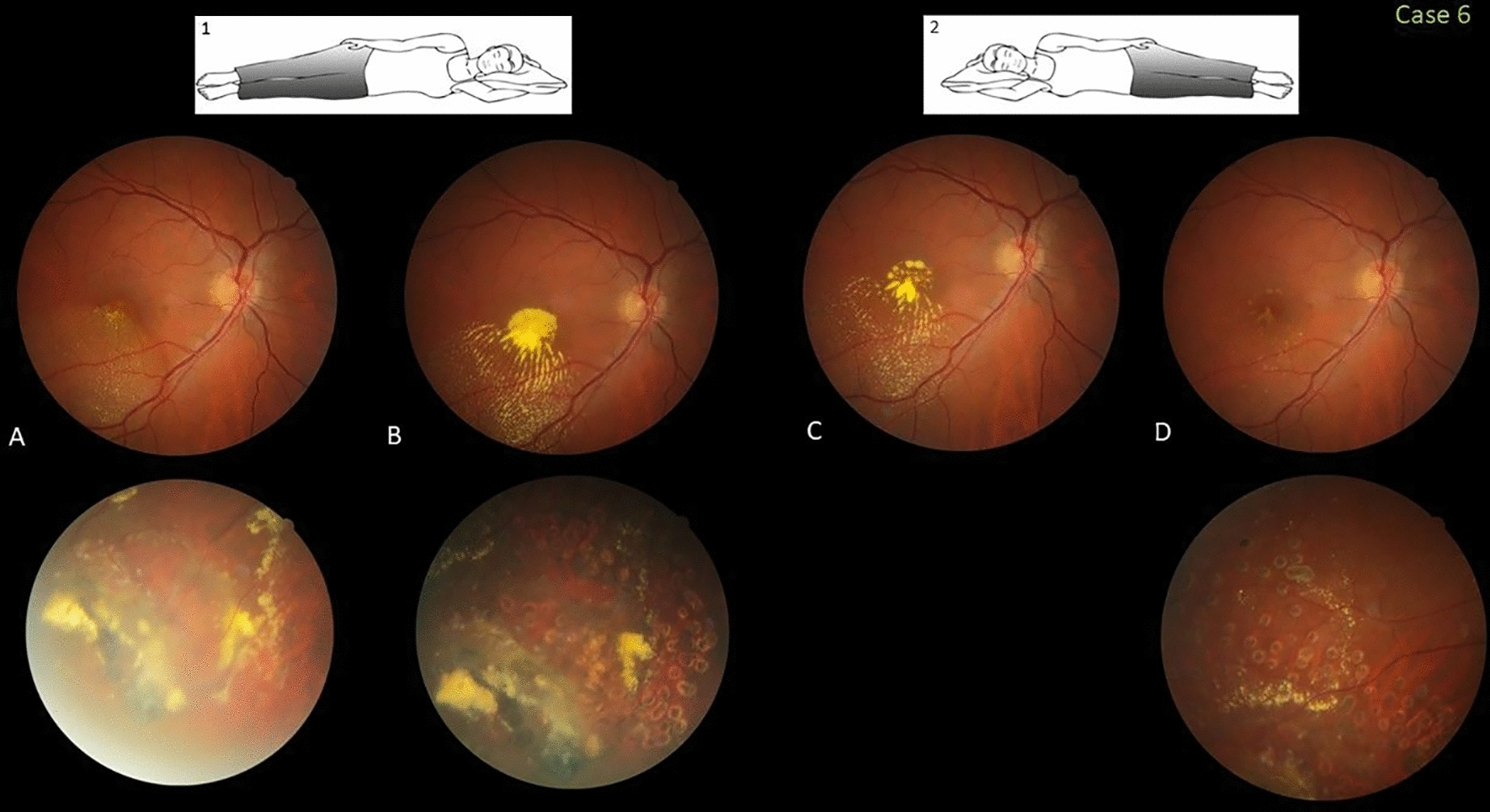
Case 6. Fovea-involving peripheral Coats disease in a 39-year-old woman who slept in the nasal-down position with a flat pillow (inset 1). **A** A color fundus image obtained when the visual acuity (VA) was 20/30 shows inferotemporal peripheral exudative retinal vascular abnormalities and an exudative retinal detachment affecting the fovea. **B** Three months after laser and sub-Tenon triamcinolone and aflibercept treatments, the VA decreased to 20/60 with pooling of concentrated exudates in the fovea. **C, D** One and 2 months after the patient adjusted the sleep position to a lateral-down high position and the head of the bed was raised (inset 2), progressive resolution of the foveal and inferior retinal exudation, respectively, is seen. The VA returned to 20/20

This healthy 39-year-old woman with previous 20/20 vision bilaterally was referred for a second opinion and management, after presenting with central visual blurring in her right eye (20/60) related to Coats disease treatment. She was taking Aromasyn (exemestane, Pfizer, New York, NY, USA) to treat breast cancer diagnosed 3 years previously. A fundus examination of the right eye showed abnormal exudative telangiectatic vessels along the temporal and inferior periphery associated with an exudative retinal detachment threatening the foveal area. Stage 2B Coats disease was diagnosed and a prompt combination of laser, injection of a sub-Tenon steroid, and three-monthly intravitreal injections of aflibercept (Eylea, Regeneron Pharmaceuticals, Tarrytown, NY, USA) were administered. After 3 months, the VA decreased to 20/60 due to pooling of concentrated exudates in the fovea (stage 2B1). The patient was advised to change from the nasal-down sleep position using a flat pillow that facilitated exudate accumulation to the temporal-down side and to raise the head of her bed. After 1 month, the foveal and inferior retinal exudates progressively resolved. The VA returned to 20/20 (Fig. 6). She was followed for recurring exudation in that eye.

### Case 7

**Fig. 7:**
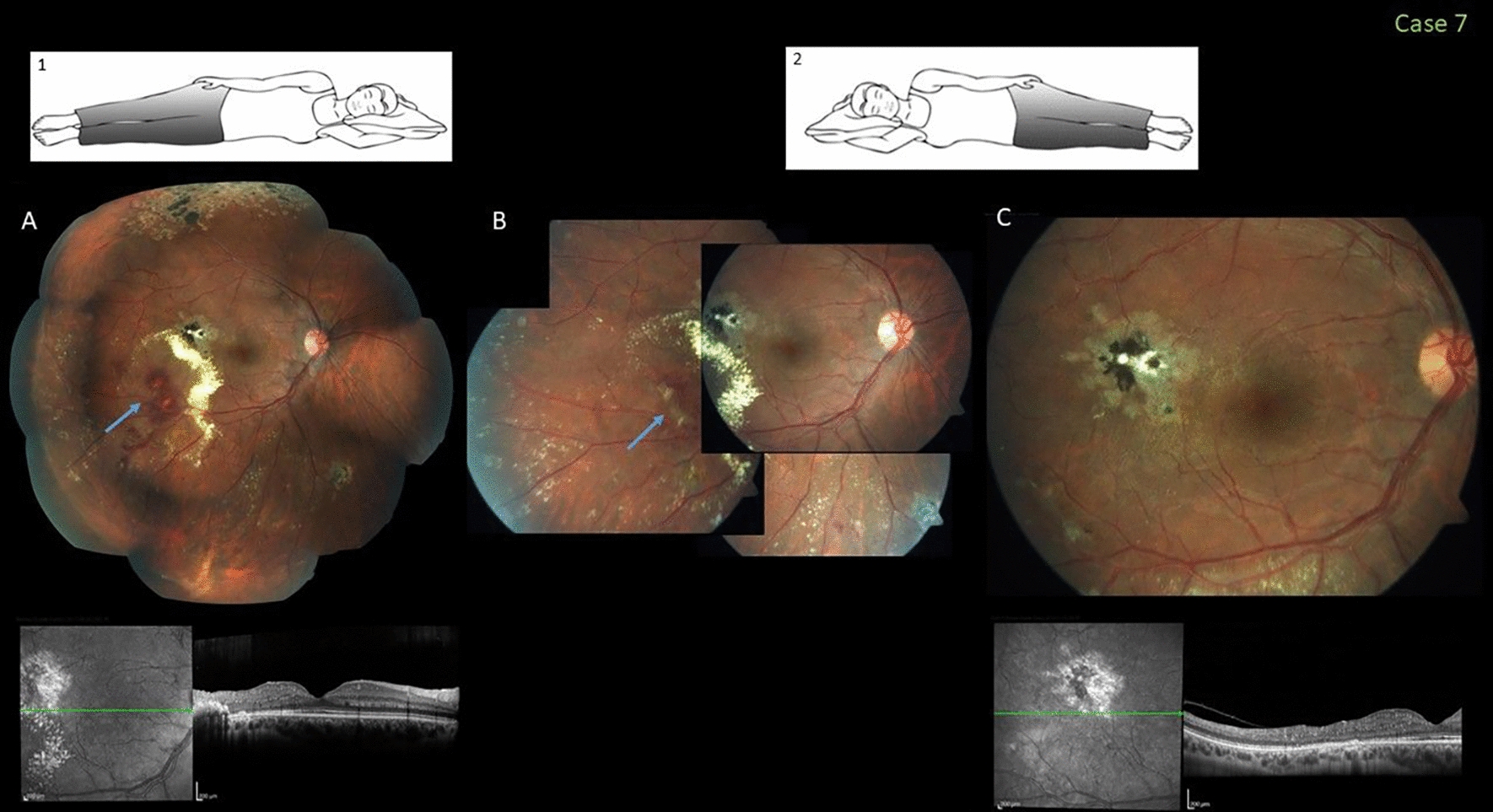
Case 7. Fovea-threatening peripheral Coats disease in a 27-year-old man who was successfully treated in the past and returned with recurring extrafoveal exudation; the visual acuity (VA) was 20/20. He adopted a nasal sleep position during this time (inset 1). **A** A color fundus shows the previous peripheral and paracentral temporal scars of laser treatment and the new site of recurring hemorrhages and exudation (arrow). A spectral-domain optical coherence tomography (SD-OCT) image confirms the presence of temporal intraretinal hyperreflective exudates. **B** Four weeks after the patient changed to a lateral-down high sleep position (inset 2) followed by laser treatment of the abnormal dilated vessels (arrow), partial dehydration of the exudates is seen. **C** Three months later, the VA improved to 20/20 and the fundus/SD-OCT images show complete resolution of the exudation

In 2017, this 27-year-old healthy man who underwent previous laser treatment for temporal peripheral Coats disease with foveal exudation in the right eye underwent a routine eye examination. He was advised regarding the sleep posture and the importance of returning for evaluations but did not do so for 3 consecutive years. At his presentation in 2019, the VA was still 20/20 and funduscopic examination showed recurring fovea-threatening exudation resulting from leaking telangiectatic and lightbulb vessels temporally. He confirmed that he resumed sleeping in the nasal-down position that facilitated exudate accumulation during this extended period of time. Considering the low risk of imminent foveal exudation in this case, the patient was again instructed to sleep in the lateral position to prevent accumulation of exudates and advised to undergo one more session of laser application (Fig. 7).

### Case 8

**Fig. 8:**
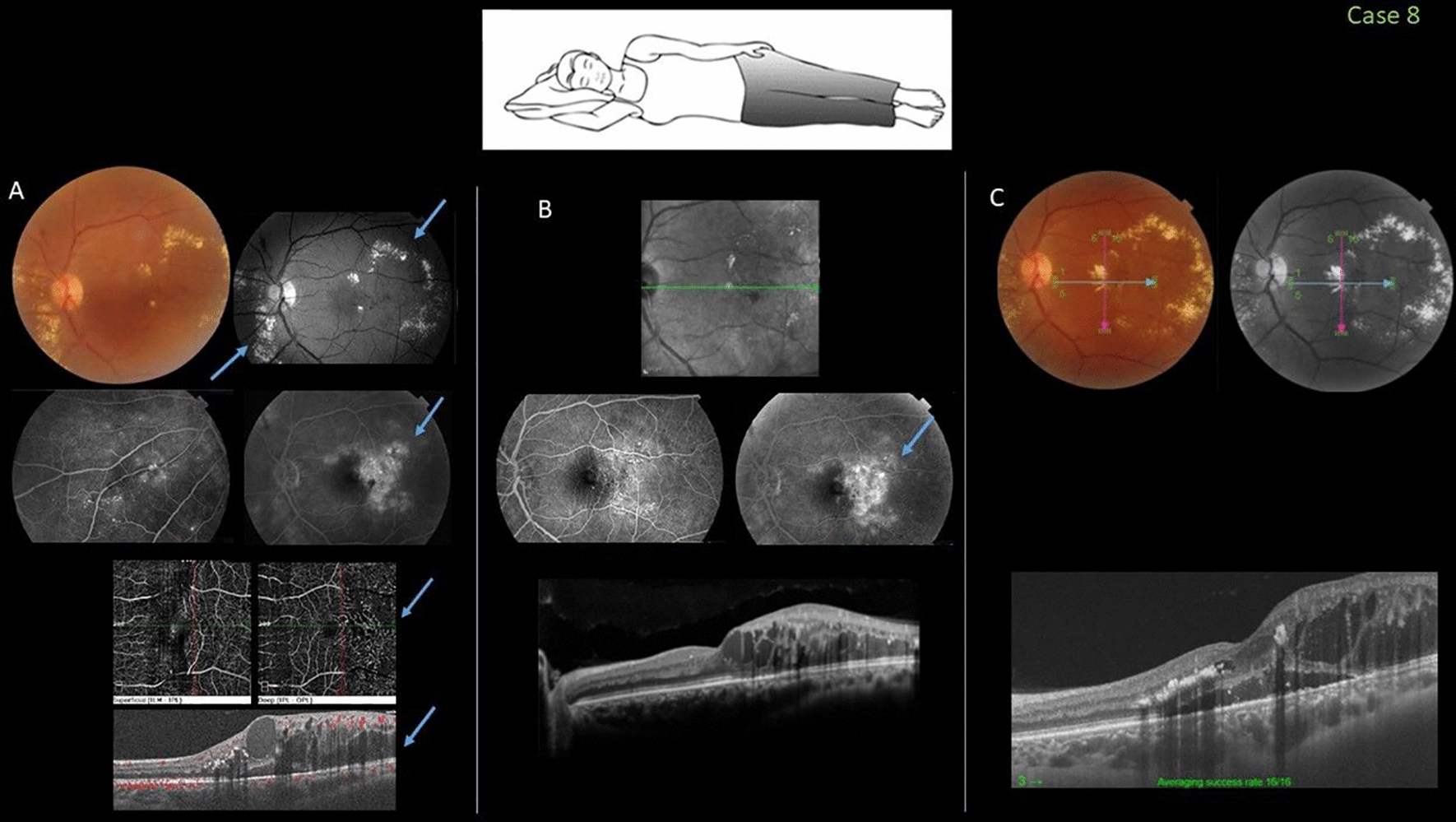
Case 8. Fovea-involving peripheral Coats-like disease in a 50-year-old man who slept in the nasal-down position before and during treatment (inset). **A** A color fundus image shows the chronic gravitational lipid exudation affecting the fovea inferonasally and inferotemporally; the visual acuity (VA) was 20/40. Near-infrared and fluorescein angiography show the areas of leakage inferonasally and around the fovea. Optical coherence tomography angiography shows the telangiectatic leaking vessels, especially at the level of the perifoveal deep capillary plexi. Structural spectral-domain optical coherence tomography confirms the presence of foveal and parafoveal edema and intraretinal hyperreflective exudates (arrows). **B** Three months after receiving monthly 1.25-mg intravitreal bevacizumab injections and two sessions of micropulse laser application, despite the partial resolution of the yellow exudates and subfoveal fluid, there are more sites of telangiectatic perifoveal vessels (arrow). **C** Despite the recommendation of changing the sleep position and using intraocular steroids, the patient returned 3 months later with VA of 20/400 and progression of exudates nasally toward the fovea

In 2019, this 50-year-old man with controlled diabetic and hypertension was referred for evaluation of a 6-month history of visual loss in the left eye associated with Coats-like disease treatment (VA, 20/40). Fundus examination of the left eye showed abnormal regional temporal and inferior nasal leakage of telangiectatic and lightbulb capillaries associated with lipid exudative precipitation affecting the inferonasal retina and temporal foveal area. Coats-like Leber’s miliary aneurysms were diagnosed and he underwent three monthly 1.25-mg intravitreal bevacizumab injections and two applications of micropulse laser. Despite partial resolution of the exudation and subfoveal fluid, the perifoveal telangiectatic vessels increased and the VA decreased to 20/100. At that time, despite the treatment recommendation that included repositioning during sleep and administration of intraocular steroids, the patient refused and returned 3 months later with 20/400 vision due to progressive exudation toward the fovea nasally (Fig. 8).

## Discussion

We believe that Coats disease is a potentially treatable retinal disease if addressed before the disease reaches the very late stages. We also recognize that some patients with late-stage disease may have preserved central vision without treatment over the long term. We present a small case series of eight patients ranging in age from 7 to 62 years with long-standing unilateral (mostly temporal) exudative peripheral Coats and Coats-like disease involving the foveal area (VA range, 20/20–20/200). Most patients were referred for a second opinion during and/or after treatment. We observed that, at least temporally, their funduscopic appearance was prone to the effects of postural gravitational forces over the course of the day and sleeping time that may interact with the peripheral exudation to maintain the fovea intact (or not). That is, standing during the day and habitual sleep posture counterbalance each other to prevent exudation from progressing from the inferior and temporal retina. Notably, despite staying hemodynamically stable for even years with preserved central vision, this is an advanced and chronic clinical stage of the disease that often goes unnoticed by the patient. Curiously, we confirmed that all current patients initially assumed an unintentionally nasal-down sleep position that favored migration of the temporal exudation toward the fovea during sleep time. This seems to be a frequent observation in patients with FTPCD, especially those presenting with a long-standing perifoveal line of lipid precipitation; that was a clinical clue that better elucidated the actual effects of the gravitational forces, protecting and/or threatening the fovea, before and after treatment, respectively. We emphasize the importance of understanding these gravitational effects on the eye before treating patients with FTPCD [8, 9] (Table 1). As a result of these retinal gravitational forces, we hypothesized that the patients respond with local compensatory overreactive physiologic forces from the unaffected retinal posterior segment (secondary dilated venous capillaries, RPE/choriocapillaris complex) during an as-yet-undetermined period of time. We believe that these reactive forces persistently drain the fluid component of the exudate, causing the larger molecules to precipitate inside the retina and away from the fovea in the plexiform and nuclear layers. This was appreciated in all of our cases, especially regarding the limits of the foveal area in cases 1 and 2, in which maximal dehydration of the exudate appears to have occurred (linear precipitation). However, some patients with previous FTPCD first presented with fovea-involving exudation (cases and Figs. 3, 4, 5, 6, and 8), which we attributed to expected decompensation of this mechanism of foveal protection due to persistent Coats disease exudation. Interesting, in these cases, we noticed that promptly changing the position during sleep time (to a temporal-down position) may, somewhat, initially protect the fovea from further exudate invasion. For example, case 4 first presented with recent fovea-involving intraretinal exudation that was successfully managed initially with the recommendation of a simple change in the sleep position for a few days (Fig. 4). However, in those cases with fovea-threatening peripheral Coats disease (VA, 20/20), despite evidence of the cause/effect gravitational forces not observed in most patients, we intend to demonstrate our thoughts and experience dealing with such critical clinical cases (cases 1 and 2). In general, there is a normal tendency to indicate prompt conventional and invasive treatment to preserve the central vision in these young patients. Furthermore, independent of the advanced clinical stage of exudation, it seems that the adopted nasal-down sleep position in cases 1 and 2 (favoring migration of the peripheral exudation toward the fovea) protected the fovea for years (VA, 20/20), and that was unnoticed before the unexpected fovea-involving exudation associated with such prompt invasive treatment (Figs. 1, 2). We believe that upright daytime and the preferred nasal sleep position in these cases make compensatory forces operate at cardinal retinal sites of maximal fluid aspiration, i.e., at the posterior retina (RPE/cc complex) and especially around the boundaries of the foveal area by the dilated 360-degree terminal ends of the retinal venous capillaries (inferior and temporal precipitation lines of heavy exudates). To explain the unexpected negative results in some patients during treatment (also observed in cases 6 and 8) we noticed that prompt and inadvertent conventional treatment may quickly decompensate these pre-installed stable compensatory mechanisms of posterior retina and foveal protection, especially during the nasal-down sleep position. We believe these patients respond with a treatment-reactive type of inflammatory intraretinal and/or subretinal fluid accumulation that might explain the sudden uncontrolled displacement of large molecules toward the central foveal area (Additional file 1). Therefore, prematurely performing an invasive intraocular procedure has the potential to cause disequilibrium of these retinal-aspirating forces and permit passive migration of secondary fluid (serous retinal detachment) and combine heavy exudates toward the foveal center. Sequential shifting of the exudation toward the nasal part of the fovea is seen often due to the remaining 180 degrees of perifoveal venous capillaries, previously aspirating maximally to maintain a dry fovea [7, 10–13]. After that, late subfoveal scarring or reactive chronically leaking perifoveal telangiectatic vessels may occur. As representative examples, cases 1 and 2 first were diagnosed with longstanding disease and 20/20 vision before treatment (Figs. 1, 2). Despite adopting an unfavorable nasal sleep position confirmed after treatment, both cases were stable with a preserved fovea and 20/20 vision before management. Unfortunately, both patients developed irreversible fibrotic foveal scars after treatment. Would these and other similar cases (case 8, lately) show better results if the patients had been instructed to reposition during sleep (from a nasal- to temporal-down posture at night for some weeks) before and after treatment? We believe so, as demonstrated in cases 3, 4, 5, 6, and 8. Since side sleeping is the most popular position, lasting hours during the night, both a temporal-down but especially a nasal-down position demonstrated the importance of the gravitational forces in controlling the fluid and exudates migrating toward the foveal area. Indeed, FTPCD cases with recent signs of initial lipid foveal deposition might also have evidence of lipid dissipation after switching the sleep position and before any intraocular management (cases 3, 5, 6). Therefore, due to chronic gravitational retinal forces in equilibrium, patients with FTCPD must be evaluated cautiously regarding treatment. Case 7, in whom it was possible to adjust the sleep repositioning together with slowly applied laser sessions, had impressive long-standing recovery of the foveal anatomy and function (Fig. 7). We believe that along with traditional treatment for Coats disease, the effect of posture should always be observed to better understand the disease physiopathology and management. Unfortunately, that was not seriously considered in some of our cases to achieve vascular stability of the fundus and preservation of central vision.

**Table 1:** Clinical features data for eight patients with FTPCD

					Management				
Patient/sex/age	Involved eye	VA at on Set	Leaking site	Sleeping position at on Set	Sleeping repositioning	Observation	Treatment	Follow up (monthos)	VA at outcome
1/77444M/7	OS	20/20	Inferior Temporal	Nasal	No	No	Laser+Antl-Vegf	2	CF
2/ F/ 11	OS	20/20	Inferior Temporal	Temporal	No	No	Laser+Antl-Vegf	6	CF
3/M/11	OS	20/100	Temporal	Nasal	Yes	Yes	Laser+Antl-Vegf	12	20/30
4/M/34	OS	20/100	Inferior Temporal	Nasal	Yes	Yes	Ozuroex	1	20/30
5/F/62	OS	20/150	Temporal	Nasal	Yes	Yes	Laser+Ozuroex	6	20/40
6/F/39	0	20/60	Inferior Temporal	Nasal	Yes	Yes	Laser + Antl-Vegf •ST STEROIO	6	20/20
7/M/27	0	20/20	Inferior Temporal	Nasal	Yes	Yes	Laser	3	20/20
8/M/50	OE	20/40	Inferior Temporal	Nasal	No	No	Laser + Antl-Vegf	3	20/400

## Supplementary Information

**Additional file 1.** Vitreoretinal surgery movie showing internal subretinal exudate drainage in a case with advanced Coats’ disease with total retinal detachment.

## Data Availability

Not applicable.
