# Correction: Fovea-threatening and fovea-involving peripheral Coats disease: effects of posture and intervention

**DOI:** 10.1186/s40942-023-00450-3

**Published:** 2023-03-09

**Authors:** Eduardo Cunha de Souza, Evandro Rosa, João Rafael de Oliveira Dias, Fernando Korn Malerbi, Bruno Campelo Leal, Helio Paulo Primiano Junior

**Affiliations:** 1 Faculdade de Medicina da, Universidade de São Paulo, Av. Dr. Arnaldo, 455, São Paulo, 01246-903 Brazil; 2 Hospital de Olhos Sadalla Amin Ghanem, Joinville, Brazil; 3 Universidade Federal de São Paulo, São Paulo, Brazil; 4 Faculdade de Medicina da Unit de Sergipe, Aracaju, Brazil

**Correction:** **International Journal of Retina and Vitreous (2022) 8:42****https://doi.org/10.1186/s40942-022-00382-4**

Following publication of the original article [1], the authors identified a typesetting error in Table 1. The corrected Table 1 is supplied in this correction article.

**Table 1:** Clinical features data for eight patients with FTPCD

					Management				
Patient/sex/age	Involved eye	VA at onset	Leaking site	Sleeping position at onset	Sleeping repositioning	Observation	Treatment	Follow up (months)	VA at outcome
1/ M/ 7	OS	20/20	Inferior temporal	Nasally down	No	No	Laser + Anti-Vegf	2	CF
2/ F/ 11	OS	20/20	Inferior temporal	Nasally down	No	No	Laser + Anti-Vegf	6	CF
3/M/11	OS	20/100	Temporal	Nasally down	Yes	Yes	Laser + Anti-Vegf	12	20/30
4/M/34	OS	20/100	Inferior temporal	Nasally down	Yes	Yes	Ozurdex	1	20/30
5/F/62	OS	20/150	Temporal	Nasally down	Yes	Yes	Laser + Ozurdex	6	20/40
6/F/39	OD	20/60	Inferior temporal	Nasally down	Yes	Yes	Laser + Anti-Vegf + ST STEROID	6	20/20
7/M/27	OD	20/20	Inferior temporal	Nasally down	Yes	Yes	Laser	3	20/20
8/M/50	OE	20/40	Inferior temporal	Nasally down	No	No	Laser + Anti-Vegf	3	20/400
